# *Neoamphitrite undevigintipes*, a new terebellid species from South Korea (Annelida, Terebellida, Terebellidae)

**DOI:** 10.3897/zookeys.943.48760

**Published:** 2020-06-22

**Authors:** Hyun Ki Choi, Hana Kim, Seong Myeong Yoon

**Affiliations:** 1 National Institute of Biological Resources, Incheon, 22689, South Korea National Institute of Biological Resources Incheon South Korea; 2 National Marine Biodiversity Institute of Korea, Seocheon, Chungcheongnam-do 33662, South Korea National Marine Biodiversity Institute of Korea Seocheon South Korea; 3 Department of Biology, College of Natural Sciences, Chosun University, Gwangju 61452, South Korea Chousun University Gwangju South Korea

**Keywords:** *
Amphitrite
*, COI, new species, Polychaeta, polychaete, systematics, taxonomy

## Abstract

A detailed description and illustrations of a new terebellid species are provided, and molecular information based on partial sequences of the mitochondrial cytochrome c oxidase subunit I (COI) gene are included. The new species, *Neoamphitrite undevigintipes***sp. nov.**, is described from the deep sea off the eastern coast of South Korea. It is similar to *Neoamphitrite groenlandica* (Malmgren, 1866) in that the thorax has 19 notopodial chaetigers. However, *Neoamphitrite undevigintipes***sp. nov.** is clearly distinguishable from *N. groenlandica* in having the uncini of the first abdominal chaetiger arranged in a single row and in having 12 ventral shields. A taxonomic key to all known *Neoamphitrite* species is also included.

## Introduction

The genus *Neoamphitrite* Hessle, 1917 is a terebellid polychaete assigned to the subfamily Terebellinae Johnston, 1846 (Fauchald 1977; Londoño-Mesa 2009). Members of this genus usually have distinct lateral lobes on anterior segments, three pairs of dichotomous branchiae, distally hirsute notochaetae, and the uncini beginning at segment 5 (Fauchald 1977; Reuscher et al. 2012). This genus has been confused with *Amphitrite* Müller, 1771 due to controversial morphological differences between the two genera (Hessel 1917; Fauvel 1927; Hutchings and Glasby 1988; Reuscher et al. 2012). Hessle (1917) considered that *Neoamphitrite* is distinguishable from *Amphitrite* by the branchial stem and nephridial papillae. *Neoamphitrite* has dichotomous branchiae with a well-developed stem and nephridial papillae with a free tube distinctly projecting from the body, while *Amphitrite* has filiform branchiae with a reduced stem and nephridial papillae with a fused tube retracted into the body. However, Fauvel (1927) and Hutchings and Glasby (1988) regarded that these differences were vague and not particularly useful in distinguishing *Neoamphitrite* from *Amphitrite*, and they considered *Neoamphitrite* to be a junior synonym of *Amphitrite*.Hessle’s (1917) classification was re-accepted and currently the genus *Neoamphitrite* is recognized in recent taxonomic works (Hilbig 2000; Londoño-Mesa and Carrera-Parra 2005; Reuscher et al. 2012). Here, the definition of *Neoamphitrite*, as described by Hessel (1917), is followed.

To date, 12 species of *Neoamphitrite* are known (Hessel 1917; Fauvel 1927; Caullery 1944; Hutchings and Glasby 1988; Hilbig 2000; Londoño-Mesa and Carrera-Parra 2005; Reuscher et al. 2012). Among the described species, *N. edwardsi* (Quatrefages, 1865), *N. ramosissima* (Marenzeller, 1884), and *N. vigintipes* (Grube, 1870), have been recorded from East Asia (Hessel 1917; Imajima and Hartman 1964; Paik 1989). While studying polychaetes from Korean waters, a new species belonging to the genus *Neoamphitrite* was found in the deep sea off the eastern coast of South Korea. In this study, a detailed description and illustrations of the new species are provided, and molecular information pertaining to the barcoded regions of mitochondrial cytochrome c oxidase subunit I (COI) gene are included. This study also includes a taxonomic key to all *Neoamphitrite* species and is based on the literature (Hessel 1917; Fauvel 1927; Caullery 1944; Hutchings and Glasby 1988; Hilbig 2000; Londoño-Mesa and Carrera-Parra 2005; Reuscher et al. 2012).

## Materials and methods

### Sampling and morphological observation

Samples were collected from the benthos of the deep sea (500–1000 m depth). Specimens were sorted using sieves with a mesh size of 0.5 mm, initially fixed with 5% formaldehyde-seawater solution, and transferred to 85% ethyl alcohol. Characteristics of the whole body were observed with appendages dissected in a petri dish using dissection forceps or surgical knives and needles under a stereomicroscope (SMZ1500; Olympus, Tokyo, Japan). Dissected specimens were mounted onto temporary slides using glycerol or permanent slides using polyvinyl lactophenol solution. Drawings were made under the stereomicroscope and light microscope (LABOPHOT-2; Nikon, Tokyo, Japan) with the aid of drawing tubes. Photographs were taken of appendages mounted on a permanent slide. Images of appendages were captured using an imaging system (LAS V4.7, Leica Microsystems, Heerbrugg, Switzerland). Specimens for scanning electron microscopy (SEM) were dehydrated using a t-BuOH freeze dryer (VFD-21S; Vacuum Device, Ibaraki, Japan). They were mounted onto stubs and coated with gold-palladium. SEM observations were conducted using a scanning electron microscope (SU3500; Hitachi, Tokyo, Japan). Type material and additional material examined were deposited at the National Institute of Biological Resources (NIBR) in Incheon, Korea and the National Marine Biodiversity Institute of Korea (MABIK) in Seocheon, Chungcheongnam-do, Korea, respectively.

### Molecular analysis

Genomic DNA was extracted from posterior segments of three specimens selected among additional materials using a DNeasy Blood and Tissue Kit (Qiagen, Hilden, Germany) according to manufacturer’s protocol. Amplifications of partial sequences of mitochondrial cytochrome c oxidase subunit I (COI) from gDNA were conducted by polymerase chain reaction (PCR) method using a set of primers: LCO 1490 5'-GGTCAACAAATCATAAAGATATTGG-3' and HCO 2198 5'-TAAACTTCAGGGTGACCAAAAAATCA-3' in COI amplification (Folmer et al. 1994). PCR amplification was conducted in a total volume of 20 µL: 10 µL of 2× DyeMIX-Tenuto (Enzynomics), 0.5 µL of each primer, 1 µL of gDNA, and 8 µL of sterile water. Touchdown-PCR was conducted according to the following cycling program: 94 °C for 5 min, 94 °C for 1 min, 50 °C for 1 min and 72 °C for 1 min, followed by 20 cycles at decreasing annealing temperatures in decrements of 0.5 °C per cycle, followed by 1 min. at 94 °C, 15 cycles of 1 min. at 40 °C, 1 min. at 72 °C, and final extension at 72 °C for 7 min. PCR products were purified with a QIAquick PCR Purification Kit (Qiagen, Chatsworth, CA, USA). Sequences for the new species were obtained with an Applied Biosystems 3730 DNA sequencer, and deposited in the GenBank under accession numbers MN306311 to MN306313. Sequences were aligned with those of other terebellid species and outgroup taxa using Geneious Pro v.9.1.8 (Biomatters, Auckland, New Zealand). Genetic distances between the new species and other species and the phylogenetic tree were produced by MEGA v.6.06 (Tamura et al. 2013).

## Systematic account

### Family Terebellidae Johnston, 1846

#### Genus Neoamphitrite

Taxon classificationAnimaliaTerebellidaTerebellidae

Hessle, 1917

74E3E0A8-8359-5A47-A157-FCE6CD79FA7C

##### Type species.

*Amphitrite affinis* Malmgren, 1866 subsequently designated by Hessle (1917).

#### Neoamphitrite undevigintipes sp. nov.

Taxon classificationAnimaliaTerebellidaTerebellidae

9A4424E8-01BD-5100-A4CA-24706979C479

http://zoobank.org/2CDD716F-CBAD-4B22-8A8A-3D51A9BB2322

Figures 1 , 2 , 3

##### Type locality.

South Korea, East Sea (Sea of Japan), 36°35'08.0"N, 130°08'19.7"E, 500–1000 m in depth.

##### Material examined.

***Holotype***: complete specimen (NIBRIV0000753905). ***Paratypes***: one complete specimen (MABIKNA00156356); one complete specimen (MABIKNA00156357); one complete specimen (MABIKNA00156358); one complete specimen (MBIKINA00156359); one complete specimen (MABIKNA00156360). ***Non-type material***: 16 specimens (all complete specimens). All materials examined were collected from the type locality, 13 April 2018 using the benthic trawl mounted on RV Tamgu 21 of National Institute of Fisheries Science (NIFS) from Korea.

##### Diagnosis.

Body with distinct thoracic and abdominal region. Tentacular lobe collar-like. Peristomium with fleshy ridge on ventral side. Upper lip distinct and undulate with free margin. Lower lip well developed, projecting forward. Buccal tentacles filiform with ventral groove. Lateral lappets present on segments 2–4, well developed on segments 2 and 3, reduced on segment 4. 12 ventral shields from segment 3. Branchiae dichotomous with distinct stalk, 3 pairs, and present on segments 2–4. Nephridial papillae small, oval on segments 3–15, located between noto- and neuropodia. Notopodia present on 19 chaetigers on segment 4. Notochaetae medially winged and distally serrated. Neuropodia beginning at segment 5. Uncini avicular, short-handled, arranged in single row on segments 5–10, in double rows of beak to beak arrangement from segments 11–22, and in single row on all abdominal segments. Dental formula MF: 4–5: 5–6: 7–8. Pygidium reduced with 10 papillae.

##### Description.

**Figure 1.**
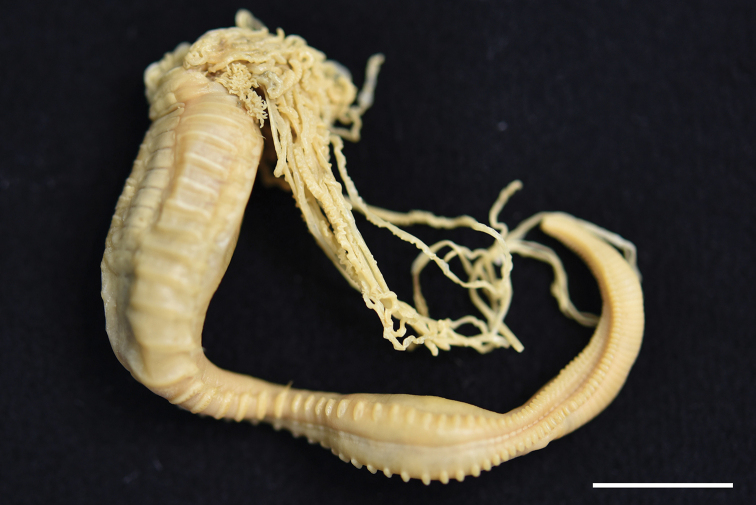
*Neoamphitrite undevigintipes* sp. nov., paratype (MABIKNA00156356), lateral view. Scale bar: 1.0 cm.

**Figure 2.**
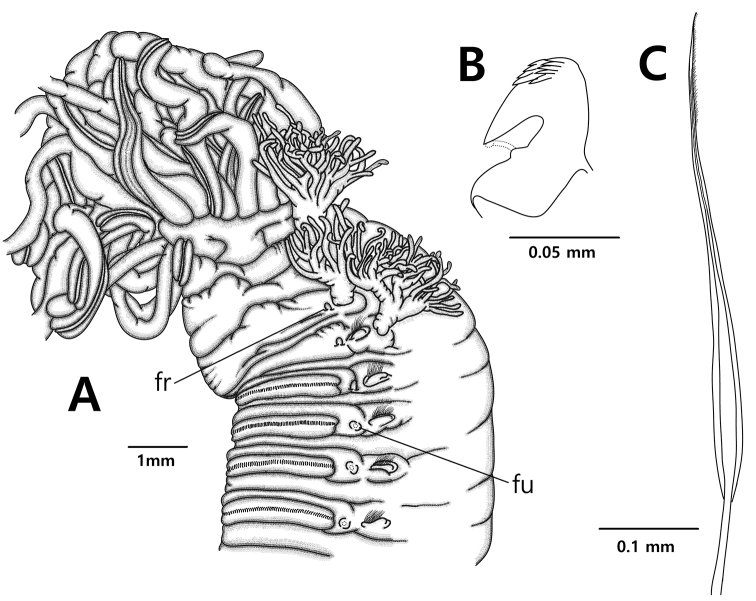
*Neoamphitrite undevigintipes* sp. nov. **A** holotype (NIBRIV0000753905) **B, C** paratype (MBIKINA00156359). **A** Anterior end, lateral view **B** uncinus **C** notochaeta. Scale bars: 1.0 mm (**A**), 0.05 mm (**B**), 0.1 mm (**C**). Abbreviations: **fr** – nephridial papillae with free tube, **fu** – nephridial papillae with fused tube.

**Figure 3.**
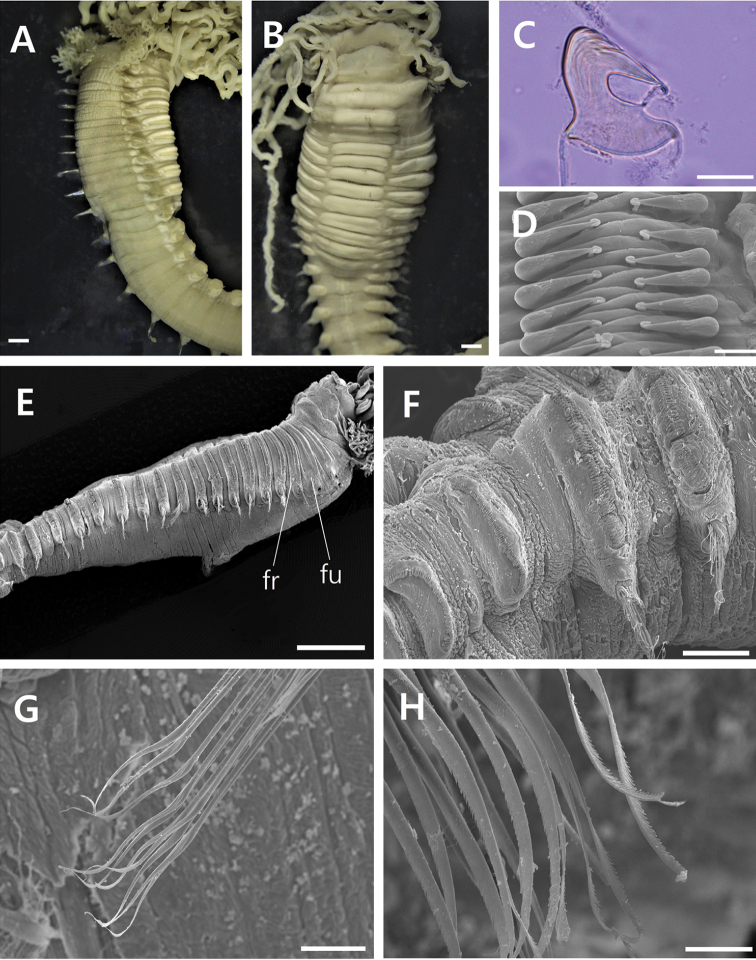
*Neoamphitrite undevigintipes* sp. nov. **A** paratype (MABIKNA00156357) **B** paratype (MABIKNA00156358) **C** (MBIKINA00156359) **D–H** (MABIKNA00156360). **A** Anterior end, lateral view **B** anterior end, ventral view **C** notopodial uncinus, lateral view **D** uncini arranged in double rows **E** thorax with 19 notopodia **F** last thoracic segment with uncini arranged in double rows and first abdominal segment with uncini arranged in single row **G** notochaetae **H** distal region of notochaetae. Scale bars: 1.0 mm (**A, B**), 0.025 mm (**C, D**), 5.0 mm (E), 0.5 mm (**F**), 0.1 mm (**G**), 0.025 mm (**H**). Abbreviations: **fr** – nephridial papillae with free tube, **fu** – nephridial papillae with fused tube.

Holotype: complete, 11.0 cm long, 1.5 cm wide at segments 10, and with approximately 74 segments. Body uniformly light beige in alcohol, without pigmentation pattern, and consist of thorax with 19 chaetigers and abdomen; anterior thoracic segments compact until about 13 and then segments slightly narrower and longer than 13 anterior segments (Figs 1, 3A, B, E). Tentacular lobe short and collar-like. Peristomium with fleshy ridge on ventral side, separated anteriorly from lower lip by groove. Upper lip distinct and undulate with free margin. Lower lip well-developed, projecting forward. Buccal tentacles filiform with ventral groove. Lateral lappets paired on segments 2–4, distinct thickness flaps, protruding forwards, and with weakly developed glandular margin; first and second lappets well-developed, but third lappets reduced in length, located on nearby base of notopodia. Branchiae paired on segments 2–4, dichotomous, with 3 tiers of branches and weakly annulated stalk distinct. Nephridial papillae small, oval, present on segments 3–15, and located between noto- and neuropodia; those with fused tube retracted into body on segments 6–8 and other with free tube distinctly projecting from the body. Ventral shields trapezoidal, broader than longer, present on segments 3–14; first shield on segment 3 with glandular margin and others with smooth margin; thereafter shields replaced by mid-ventral groove extending to pygidium (Figs 2A, 3A, B, E). Notopodia short, rectangular, present on segments 4–22 (chaetigers 1–19); last 2 or 3 pairs becoming much shorter. Notochaetae slightly curved, medially winged and distally serrated, types of 2 lengths; chaetae on anterior row at least half as long as those on posterior row (Figs 2C, 3E–H). Neuropodia beginning from segment 5 as low rectangular ridges, and with uncini arranged in single rows on segments 5–10 (chaetigers 2–7), uncini in double rows beak to beak arrangement on segments 11–22 (chaetigers 8–19), and in single row on all abdominal segments. Uncini avicular, short-handled with short triangular heel, distally pointed prow, minute dorsal button, and 5 rows of secondary teeth on main fang with subrostral guard. Dental formula MF: 4–5: 5–6: 7–8 (Figs 2B, 3C, D). Pygidium reduced with encircling 10 papillae.

##### Etymology.

A combination of the Latin *undeviginti* and *pes.* This name means ‘nineteen feet’, referring to the 19 pairs of notopodia on the thoracic segments.

##### Habitat.

This species is found on the soft bottom of deep waters (500–1000 m depth) and lives in a mud tube.

##### Remarks.

In *Neoamphitrite* taxonomy, the number of notopodia is a key character for the identification of species (Hutchings and Glasby 1988; Hilbig 2000; Londoño-Mesa and Carrera-Parra 2005; Reuscher et al. 2012). *Neoamphitrite undevigintipes* sp. nov. has 19 pairs of notopodia on the thoracic segments regardless of body size and number of segments. In this respect, the new species is most similar to *Neoamphitrite groenlandica* (Malmgren, 1866), which was originally described from the Atlantic Ocean and also has 19 pairs of notopodia (Malmgren 1866; Hessle 1917; Fauvel 1927). However, the new species is clearly differentiated from *N. groenlandica* by two characteristic features. The uncini in the first abdominal chaetiger are arranged in a single row in *N. undevigintipes* sp. nov., but in double rows in *N. groenlandica*, and the new species has 12 ventral shields, but *N. groenlandica* has 14 (Malmgren 1866; Hessle 1917; Fauvel 1927).

In East Asia, three *Neoamphitrite* species, *N. edwardsi* (Quatrefages, 1865), *N. ramosissima* (Marenzeller, 1884), and *N. vigintipes* (Grube, 1870) from Japan, have been recorded (Hessle 1917; Imajima and Hartman 1964; Paik 1989). *Neoamphitrite undevigintipes* sp. nov. shows several differences from these species as follows: notopodia of *N. undevigintipes* sp. nov. are present on 19 chaetigers, compared to 17 in *N. ramosissima* and *N. edwardsi*. *Neoamphitrite undevigintipes* sp. nov. has uncini arranged in a single row in all abdominal chaetigers, while *N. vigintipes* has the uncini arranged in double rows in abdominal chaetigers except for some final chaetigers. The new species has 13 pairs of nephridial papillae, whereas six, nine, and 12 pairs are present in *N. ramosissima*, *N. edwardsi*, and *N. vigintipes*, respectively (Hessle 1917; Imajima and Hartman 1964; Paik 1989).

Hessle (1917) suggested that *Neoamphitrite* species are distinguished from *Amphitrite* species by having nephridial papillae with free tubes distinctly projecting from the body rather than fused tubes retracted into the body. However, in several specimens of *N. undevigintipes* sp. nov., the nephridial papillae have fused tubes in two or three of all nephridial papillae pairs. Hutchings and Glasby (1988) mentioned that the form of nephridial papillae is difficult to use as a generic diagnostic feature because it can be variable according to the state of specimens. This character was overlooked in diagnoses of the terebellid genera (Fauvel 1927; Caullery 1944; Fauchald 1977; Hilbig 2000; Hutchings and Glasby 1988; Londoño-Mesa and Carrera-Parra 2005; Reuscher et al. 2012). In this respect, we think that the form of nephridial papillae is not yet a useful diagnostic character and that its taxonomic value should be re-examined in detail using as many species as possible. We provide a key to the species presently regarded as members of *Neoamphitrite*.

##### Genetic information.

**Figure 4.**
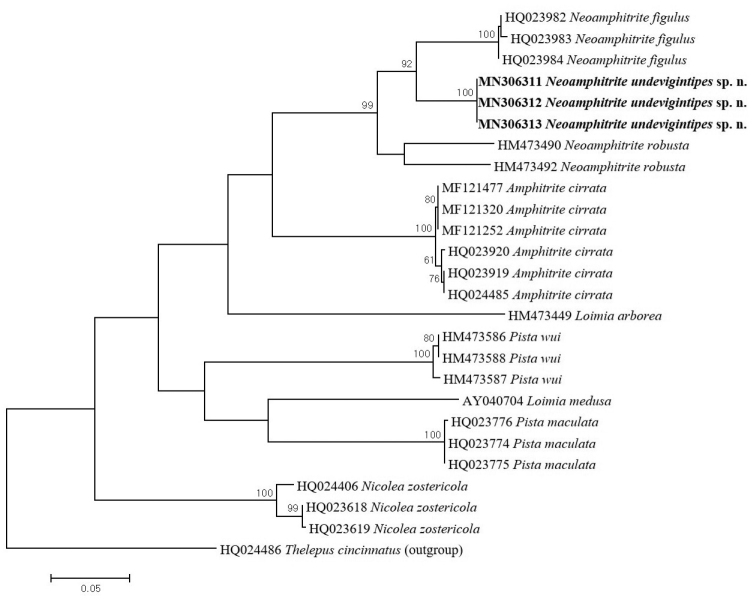
Maximum likelihood (ML) tree showing phylogenetic relationship based on COI sequences of three *Neoamphitrite* species with seven related species assigned to other genera and a outgroup species. Numbers above the branch indicate ML bootstrap values from 1000 replication.

In this study, partial COI sequences, each measuring 658 bp, were obtained from three specimens for genetic analysis of *Neoamphitrite undevigintipes* sp. nov. They are deposited in the GenBank under accession numbers MN306311 to MN306313. All COI sequences obtained were identical. Using data available from the GenBank (Carr et al. 2011; Siddall et al. 2011; Telfer and Dewaard 2017), we genetically compared the new species with two *Neoamphitrite* species, *N. figulus* (Dalyell, 1853) and *N. robusta* (Johnson, 1901), as well as six species belonging to the other terebelline genera: *Amphitrite cirrata* Müller, 1776, *Nicolea zostericola* Örsted, 1844, *Pista maculata* (Dalyell, 1853), *Pista wui* Safronova, 1988, *Loimia arborea* Moore, 1903, and *Loimia medusa* (Savigny, 1822). *Thelepus cincinnatus* (Fabricius, 1780) was used as the outgroup. GenBank accession numbers are represented in Table 1. Inter-specific genetic distances between the new species and two *Neoamphitrite* species, as measured by Kimura-2-parameter model, were distinct and ranged from 9.2 to 13.7%. The genetic distances between the new species and the six species in other genera ranged from 21.8 to 29.9%. In the maximum likelihood (ML) tree based on these genetic data (Fig. 4), all terebellid species showed specific validity. The new species was contained in a clade with *N. figulus* and *N. robusta*. At the generic level, the *Neoamphitrite* clade, including the new species, was closely related to *A. cirrata*, agreeing with the taxonomic view that *Neoamphitrite* and *Amphitrite* share many morphological features except for differences in the morphology of branchiae (Hessel 1917; Fauvel 1927; Hutchings and Glasby 1988; Reuscher et al. 2012). However, *Neoamphitrite* was monophyletic and clearly distinguishable from *A. cirrata* in the ML tree, supporting the known morphological differences between two genera (Hessel 1917; Fauvel 1927; Hutchings and Glasby 1988; Reuscher et al. 2012). Despite our results, further genetic studies with additional data and including more species of *Neoamphitrite* and *Amphitrite* are needed to confirm the phylogenetic relationship between the two genera.

**Table 1.** GenBank accession numbers for COI sequences obtained in the present study.

Species	Genbank accession number	Data source
*Neoamphitrite undevigintipes* sp. nov.	MN306311–MN306313	Present study
*Neoamphitrite figulus*	HQ023982–HQ023984	Carr et al. 2011
*Neoamphitrite robusta*	HM473490, HM473492	Carr et al. 2011
*Amphitrite cirrata*	HQ023919, HQ023920, HQ024485	Carr et al. 2011
	MF121320, MF121320, MF121477	Telfer and Dewaard 2017
*Nicolea zostericola*	HQ023618, HQ023619, HQ024406	Carr et al. 2011
*Pista maculata*	HQ023774–HQ023776	Carr et al. 2011
*Pista wui*	HM473586–HM473588	Carr et al. 2011
*Loimia arborea*	HM473449	Carr et al. 2011
*Loimia medusa*	AY040704	Siddall et al. 2011
*Thelepus cincinnatus* (outgroup)	HQ024486	Carr et al. 2011

### Key to known species of the genus *Neoamphitrite* Hessle, 1917

**Table:** 

1	Notopodia present on first 15 chaetigers	***N. hydrothermalis* Reuscher, Fiege & Wehe, 2012**
–	Notopodia present on more than first 15 chaetigers	**2**
2	Notopodia present on first 17 chaetigers	**3**
–	Notopodia present on more than first 17 chaetigers	**7**
3	Thoracic neuropodial tori extending to ventral shield or mid-ventral groove	**4**
–	Thoracic neuropodial tori not extended	**5**
4	Nephridial papillae on segments 3–11	***N. robusta* (Johnson, 1901)**
–	Nephridial papillae on segment 3 only	***N. sibogae* (Caullery, 1944)**
5	Segments 4 with small lateral lappets	**6**
–	Segments 4 without lateral lappets	***N. ramosissima* (Marenzeller, 1884)**
6	Nephridial papillae 6 pairs on segments 3–8	***N. affinis* (Malmgren, 1866)**
–	Nephridial papillae 9 pairs on segments 3–11	***N. edwardsi* (Quatrefages, 1865)**
7	Thorax with 39 notopodial chaetigers; lateral lappets on segment 2 inconspicuous	***N. glasbyi* Londoño-Mesa & Carrera-Parra, 2005**
–	Thorax with less than 30 notopodial chaetigers; lateral lappets on segment 2 conspicuous	**8**
8	Abdominal neuropodial tori with uncini arranged in double rows present on almost all chaetigers	***N. vigintipes* (Grube, 1870)**
–	Abdominal neuropodial tori with uncini arranged in double rows absent, or present on first and second abdominal chaetigers only	**9**
9	Notopodia present on 19 thoracic chaetigers	**10**
–	Notopodia present on more than 20 thoracic chaetigers	**11**
10	First abdominal chaetiger with uncini arranged in single row	***N. undevigintipes* sp. nov.**
–	First abdominal chaetiger with uncini arranged in double rows	***N. groenlandica* (Malmgren, 1866)**
11	Nephridial papillae 7 pairs	***N. pachyderma* (Hutchings & Glasby, 1988)**
–	Nephridial papillae at least 10 pairs	**12**
12	Ventral shields 13; nephridial papillae 10 pairs	***N. grayi* (Malmgren, 1866)**
–	Ventral shields 14; nephridial papillae 12 pairs	***N. figulus* (Dalyell, 1853)**

## Supplementary Material

XML Treatment for Neoamphitrite

XML Treatment for Neoamphitrite undevigintipes
